# Genetic diversity analysis and core germplasm bank construction in cold resistant germplasm of rubber trees (*Hevea brasiliensis*)

**DOI:** 10.1038/s41598-024-65464-9

**Published:** 2024-06-24

**Authors:** Maoju Tian, Wenxiu Li, Ping Luo, Junjun He, Hualin Zhang, Qing Yan, Yanna Ye

**Affiliations:** 1 Zhanjiang Experimental Station, Chinese Academy of Tropical Agricultural Sciences/Zhanjiang Rubber Forest Economic Engineering Technology Research Center, Zhanjiang, China; 2 South Asia Tropical Crop Research Institute, Chinese Academy of Tropical Agricultural Sciences, Zhanjiang, China; 3 College of Tropical Crops, Yunnan Agricultural University, Pu’er, China

**Keywords:** *Hevea brasiliensis*, SNP marking, Genetic diversity, Fingerprint, Core collection, Group structure, Principal component analysis, Molecular biology, Plant sciences

## Abstract

The rubber tree, *Hevea brasiliensis* (Willd. ex Adr. de Juss.) Muell. Arg., is the sole plant worldwide utilized for the commercial production of natural rubber. Following years of breeding, there exists a wide array of germplasm differentiation in rubber trees. The exploration of diversity and population structure within rubber tree germplasm resources, alongside the establishment of core germplasm resources, is instrumental in elucidating the genetic background and facilitating the effective utilization and management of these resources. By employing SNP molecular marker technology, 195 rubber tree resources were amplified, their genetic diversity analyzed, and a fingerprint map was subsequently constructed. Through this process, the cold-resistant core germplasm of rubber trees was identified. The results revealed that the PIC, He, and pi values ranged from 0.0905 to 0.3750, 0.095 to 0.5000, and 0.0953 to 0.5013, respectively. Both group structure analysis and cluster analysis delineated the accessions into two groups, signifying a simple group structure. A core germplasm bank was established with a sampling ratio of 10%, comprising 21 accessions divided into two populations. Population G1 consists of 20 accessions, while population G2 comprises 1 accession. The research findings have led to the creation of a molecular database that is anticipated to contribute to the management and subsequent breeding applications of rubber tree accessions.

## Introduction

The rubber tree, a perennial tropical rainforest species belonging to the genus Hevea of the family Euphorbiaceae, originates in the Amazon River region of Brazil, South America, where it thrives in the warm and humid climate. Best known for its production of natural rubber, which has numerous industrial and commercial applications^1^, the rubber tree was first introduced to China in the early twentieth century and began to be widely planted in the 1960s. Since then, China has become one of the major rubber-producing countries in the world, leading to the development of different types of rubber trees and resulting in a diverse and rich variety of resources.

To understand the genetic differences between these types and select outstanding parents for hybrids, various researchers have analyzed the genetic diversity of different rubber tree groups, including wild germplasm^2^, cultivated germplasm^3^, breeding varieties^4^, and hybrid offspring populations^5^. In order to evaluate the genetic differences between different accessions, select outstanding hybrid parents, and exert the role of hybrid vigor, different researchers have previously analyzed the genetic diversity of different types of rubber trees, such as Weikehan wild germplasm, cultivated germplasm, breeding varieties, and hybrid offspring populations. However, as China is not a traditional planting area and often suffers from low temperature damage, there's a need to breed varieties that can adapt to these conditions. Researchers have evaluated cold-resistant accessions or varieties in natural or simulated low-temperature environments^6,7^. Yet, genetic diversity analysis of cold-resistant germplasm populations remains limited, requiring further research.

Cold-resistant germplasm resources of rubber trees are the important material basis for breeding new cold-resistant varieties and studying genetic background of rubber trees. It is very important for breeders to clarify the genetic relationship between germplasm resources and breeding materials^8^. Phenotype-based methods have been used in the past^9^, but they are prone to human error and environmental factors can cause significant deviations in analysis results. Therefore, molecular marker technology has become an important tool for constructing core germplasm banks. It provides stable and repeatable results, making it suitable for genetic diversity analysis and core germplasm bank construction research. Among them, the single nucleotide polymorphisms (SNP) markers are widely distributed on chromosomes, offering numerous advantages like rich polymorphism and genetic stability^10^. These markers are frequently utilized in genetic diversity analysis^11^, core germplasm bank construction^12^, genetic map creation^13^, and variety identification research^14^. Currently, they are the most significant markers in molecular breeding applications.

The construction of a core germplasm bank for rubber trees represents the genetic diversity of the entire population with the minimum number of varieties. This bank can improve the management and utilization efficiency of accessions^15^. Since rubber trees require a lot of land for preservation due to their size, it's crucial to use limited land to preserve as many accessions as possible while maintaining genetic diversity. Various molecular markers have been used, such as An et al.^16^ hierarchical clustering method for wild rubber trees, Fang et al.^17^ analysis of 289 resources for 27 Weikehan accessions, and Livia et al.^2^ resource analysis using 13 primers on 1117 materials. However, up to now, there is no report on the construction of core collection bank based on SNP. Because of cold resistance has always been an important breeding goal for rubber tree varieties in China due to its colder winters. Long breeding cycle and limited cold-resistant germplasm resources lead to great difficulty in breeding. Hence, there is an urgent need to develop a fast, precise, and straightforward method for enhancing the identification and breeding efficiency of cold-resistant rubber tree germplasm resources. SNP detection offers the benefits of speed and accuracy, making it an ideal technique for identifying the core bank of cold-resistant germplasm resources in rubber trees, thereby expediting molecular genetic breeding.

To better understand the genetic diversity of 196 rubber tree cold-resistant germplasm, this study employed SNP molecular markers. This approach allowed for early and accurate identification of cold resistance in the field. Subsequently, a fingerprint map was created and a core library of cold-resistant rubber tree accessions was established. The main objective of this research was to analyze the genetic differences among various germplasm, preserving the maximum genetic diversity of rubber germplasm resources. This would ultimately enhance the efficiency of hybrid breeding selection. Furthermore, it would provide a theoretical framework for managing and utilizing cold-resistant germplasm resources in rubber trees.

## Results and analysis

### Genetic diversity analysis of SNP primers

In the study, 99 SNP loci were successfully amplified from 195 rubber tree samples using 40 pairs of primers (Supplementary Table 6). The value of *PIC* range from 0.0905 to 0.3750, with an average of 0.2403. The marker with the highest *PIC* value is locus the 89th locus marker, attaining a *PIC* of 0.3750. The *He* statistic, which measures genetic diversity, ranges from 0.0950 to 0.5000, with an average of 0.2934. Similarly, the *pi* statistic, which estimates nucleotide diversity, spans from 0.0953 to 0.5013, with an average of 0.2942. Notably, SNPs 37, 57, 54, and 71 exhibited deletions, SNPs 68 and 99 showed insertions, and the remaining loci exhibited mutations. Overall, The average values of *PIC*, *He*, and *pi* did not exceed 0.3, indicating that the genetic diversity of cold resistant germplasm in rubber trees in this study is not high.

### Population structure, cluster analysis, and PCA

**Figure 1:**
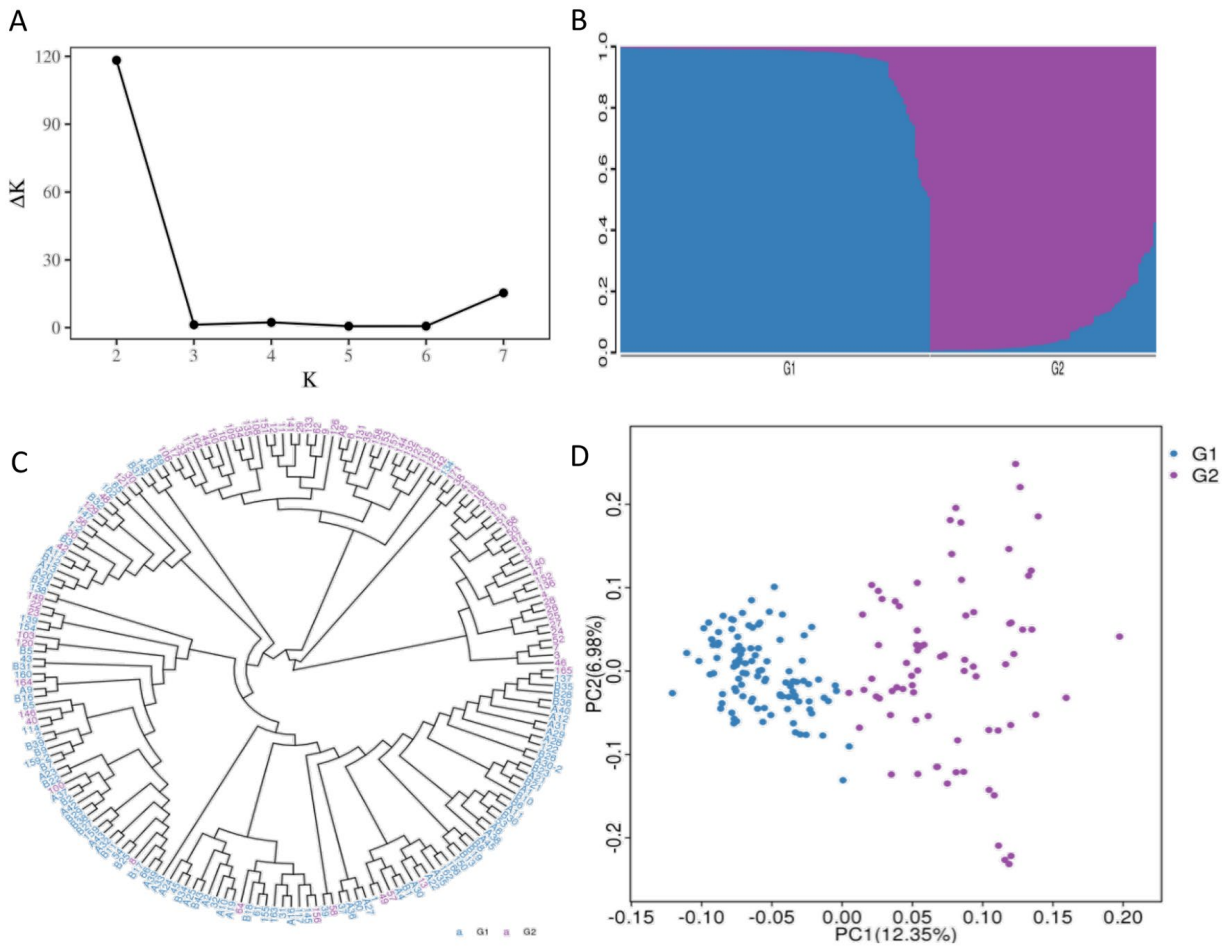
Population structure of the accessions. Ad hoc quantity (ΔK) confirmed a higher likelihood at K = 2 (**A**). Genetic structure of 195 rubber tree resources based on SNP markers (**B**). Cluster analysis of 195 rubber trees (**C**). Principal component analysis of 195 rubber trees (**D**).

By employing group structure analysis, cluster analysis, and PCA, 195 materials were classified into two groups. The genetic structure of the test material population underwent analysis using the STRUCTURE software, and the optimal K value was determined using the methods of Evanno et al^18^. In Fig. 1A, the variation curve of K with increasing K value is illustrated, demonstrating a clear peak at K = 2, indicating that dividing the materials into two subgroups is more reasonable and highlights the genetic structure. Furthermore, Fig. 1B portrays the population genetic structure of 195 rubber tree resources, suggesting that dividing them into two groups is more suitable, with minimal gene exchange among most individuals, and only a small number experiencing gene exchange.

The neighbor-joining method in MEGA7 software was utilized to construct a phylogenetic tree, and the clustering analysis of 195 materials was visualized using ggtree to draw a clustering graph. The results revealed that the 195 materials were divided into two major groups: the first group included 169 materials, while the second group comprised 26 materials (Fig. 1C). Furthermore, the analysis indicated limited differentiation among the groups, suggesting that the degree of variation in the analyzed germplasm resources is not significant.

The PCA results for 195 rubber tree resources are presented in Fig. 1D, showcasing the contribution rates of PC1 and PC2 at 12.35% and 6.98% respectively. It is evident that the distribution of population G1 is relatively concentrated, while the distribution of population G2 is relatively scattered. This observation indicates that the genetic relationship within population G1 is close, whereas the genetic relationship of population G2 is relatively distant. Additionally, the similarity of population G1 is higher than that of population G2, with only a small number of genotype crossovers between the two populations. These principal component results align with those of clustering.

Furthermore, the genetic diversity of the rubber tree classification population was assessed, the mean values of *He*, *PIC*, and *pi* in the tested rubber tree populations are 0.2820, 0.2296, and 0.2813, respectively (Table 1). Notably, the values of *He*, *PIC*, and *pi* in G2 surpass those in G1. Both the PCA results and genetic diversity analysis align, confirming that the genetic diversity of population G2 exceeds that of G1.

**Table 1:** Genetic diversity parameters of 195 rubber trees.

Population	*He*	*PIC*	*pi*
G1	0.2535	0.2059	0.2547
G2	0.3104	0.2533	0.3123
Mean	0.2820	0.2296	0.2835

### Construction of core germplasm bank

After using five different sampling ratios in combination with clustering analysis to establish a core germplasm bank, we determined an optimal preservation ratio of 10% for this batch of germplasm, effectively upholding the diversity of the original population. The results, presented in Table 2, indicate that when the sampling ratio of population G1 ranged from 10 to 50%, the expected values for *He*, *PIC*, and *pi* varied from 0.2674 to 0.2928, 0.2179 to 0.2368, and 0.2710 to 0.3015, respectively. A comparison with the diversity coefficient of the original population revealed a significant increase in diversity across varying sampling ratios, particularly at the 10% and 40% sampling ratios for expected *He*, *PIC*, and *pi*. Pursuant to the principle of minimizing the number of core germplasms while conserving the genetic diversity of the original population, we recommend a sampling ratio of 10%.

**Table 2:** Genetic diversity parameters of rubber tree core germplasm bank constructed with different sampling ratios.

	Sampling ratio/%	Number	*He*	*PIC*	*pi*
G1	10	18	0.2899	0.2357	0.3015
	20	35	0.2781	0.2253	0.2836
	30	51	0.2674	0.2183	0.2710
	40	67	0.2928	0.2368	0.2958
	50	81	0.2711	0.2179	0.2746
	100	169	0.2535	0.2059	0.2547
G2	10	3	0.3277	0.2643	0.3503
	20	5	0.3042	0.247	0.3168
	30	8	0.3262	0.2643	0.3348
	40	10	0.3212	0.2592	0.3279
	50	12	0.3313	0.2678	0.3347
	100	26	0.3104	0.2533	0.3123

When considering population G2, the expected values for *He*, *PIC*, and *pi* varied from 0.3042 to 0.3313, 0.247 to 0.2678, and 0.3168 to 0.3503, respectively, as the sampling ratio ranged from 10 to 50%. In general, the diversity fluctuated across different sampling ratios in comparison to the original population. Notably, the *PIC* exhibited relatively high values at 10% and 50% sampling ratios. As a result, we recommend a sampling ratio of 10% for population G2.

Comprehensive analysis indicates that the rubber tree germplasm population selected with a 10% sampling ratio fully reflects the genetic diversity of the original population and can be confirmed as the core germplasm bank of rubber trees.

**Figure 2:**
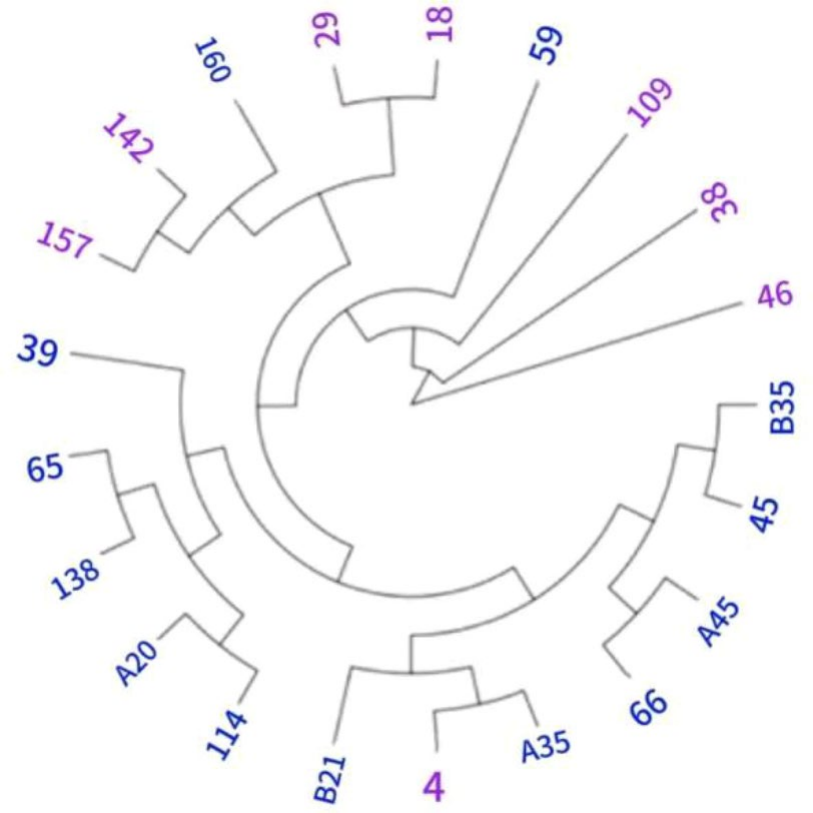
Cluster graph with a proportion of 10%. G1 is represented in blue (**a**), while G2 is represented in purple (**a**).

At a 10% sampling ratio, a population clustering map (Fig. 2) was created, revealing two distinct major groups that mirrored the original population. Group G1 comprises 20 germplasms, while Group G2 consists of only one germplasm, labeled as germplasm 46.

### Construction of fingerprint spectra

**Figure 3:**
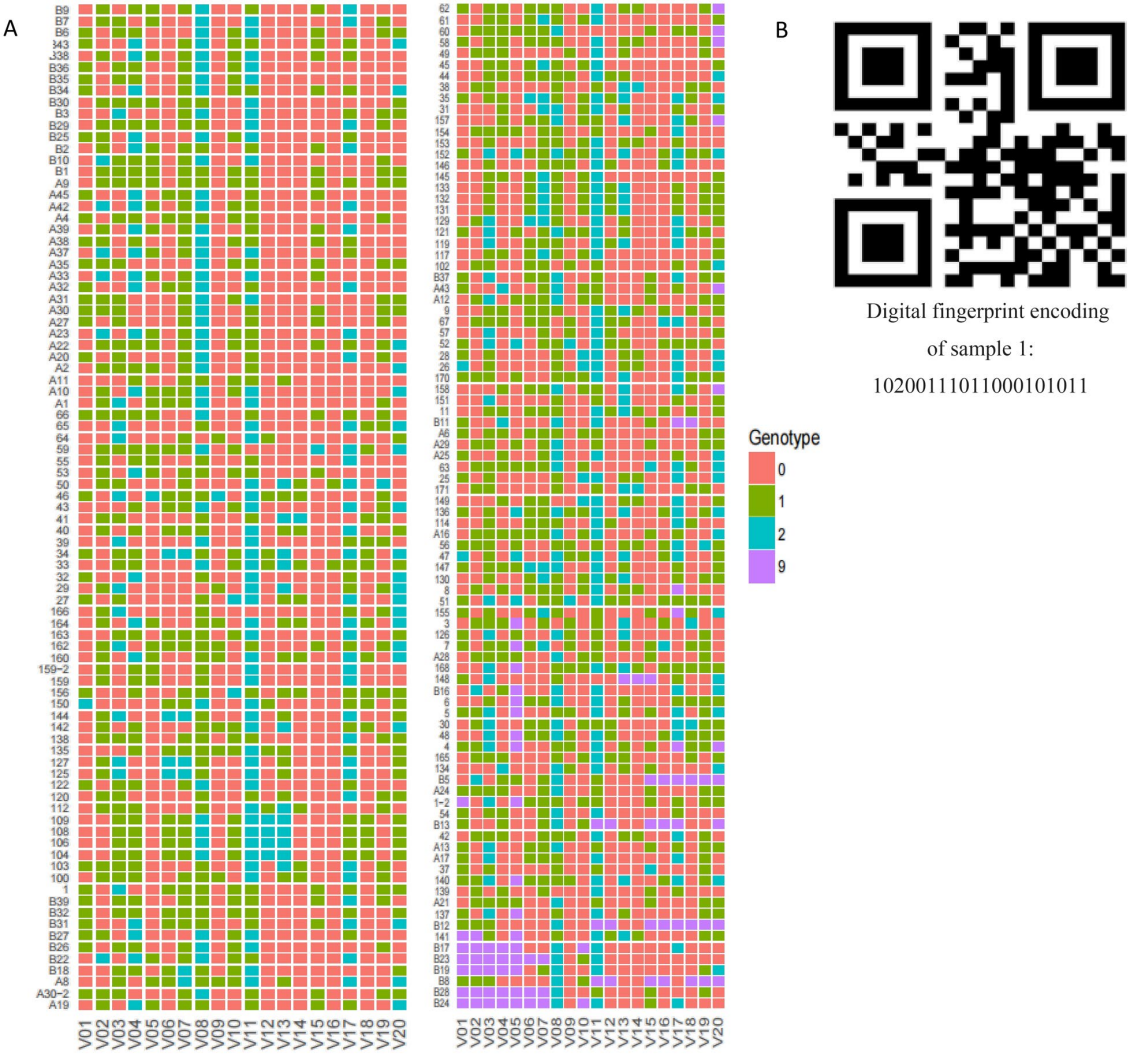
The heatmap information of 195 rubber tree (**A**). One of the material QR codes and digital fingerprint encoding (**B**).

Fingerprint maps play a crucial role in identifying and distinguishing varieties. In this experiment, 20 highly differentiated SNPs were chosen to create a fingerprint, from which a heat map was produced (refer to Fig. 3A for the heatmap information). From the heat map, it can be seen that although a small portion of accessions has unrecognizable codes (represented in purple), all materials can be distinguished. The genotype was converted into a digital fingerprint code, such as 10,200,111,011,000,101,011, and then utilized to generate a QR code, forming a DNA fingerprint map. Sample 1 (Fig. 3B) serves as an illustrative example.

## Discussion

### Genetic diversity analysis

The genetic diversity of cold-resistant rubber tree germplasm resources in this study is found to be relatively low, with limited differentiation. In comparison, Ahasanul et al.^19^ analyzed the genetic diversity of 350 flax germplasms using 6200 SNP markers, revealing an *He* range of 0.08–0.53 and *PIC* range of 0.07–0.4728. Similarly, Du Heshan et al.^20^ employed 92 SNPs to examine the polymorphism of 271 chili varieties, yielding an average *PIC* and *He* values of 0.31 and 0.429, respectively. Additionally, Wirulda et al.^21^ investigated the genetic diversity of 37 rubber tree germplasms using 33 pairs of SNP markers, identifying *PIC* range of 0.0963—0.5135. Contrastingly, this study assessed the genetic diversity of 195 cold-resistant rubber tree resources using 40 SNP primers, and found *PIC* values ranging from 0.0905 to 0.3750, with a mean of 0.2403, the values of *He* ranging from 0.095 to 0.5000, with a mean of 0.2934, and the value of *pi* ranging from 0.0953 to 0.5013, with a mean of 0.2942. There are three reasons for the relatively low genetic diversity in this study. Firstly, the domestication history of rubber trees, which have only been cultivated for over a century. Secondly, the limited source of species, mainly the Weikehan variety, possibly contributed to the decrease in genetic diversity^22^. Thirdly, it may be due to the double allele nature of SNP^23^. In the future, we should select more cold-resistant germplasm and use wild germplasm resources for hybridization and improvement to expand the genetic diversity of cold-resistant germplasm of rubber trees.

### Group structure analysis, cluster analysis, and principal component analysis

The group classification results obtained from the analysis are considered both reasonable and reliable. In this study, a population structure analysis, cluster analysis, and principal component analysis were conducted on 195 rubber tree materials using Structure software, MEGA7 software, and PLINK software. The group structure was established based on a mixed model of Markov Chain Monte Carlo (MCMC) algorithm and Bayesian framework, aiming to minimize the impact of human factors on group division and ensuring high accuracy^12^. The cluster analysis involved the construction of evolutionary trees using the adjacency method^24^, which is particularly suitable for short sequences with small evolutionary distances and limited information sites. Given the parameters of this experiment, the adjacency method was found to be the most suitable. Principal component analysis, a statistical method for condensing multiple indicators into a smaller set of comprehensive indicators, was also employed^25^. The combination of these three methods consistently indicated the division of the rubber tree resources into two major groups, demonstrating the reasonableness of the classification and its significance in understanding the genetic relationships between rubber tree populations.

Compared with SSR clustering results of rubber tree population, there are fewer clustering groups. For example, Li Wenxiu used UPGMA to cluster rubber tree hybrids, which can be divided into three groups. Long Qingyi used UPGMA to cluster 289 rubber tree Weikehan germplasm and divided them into three major groups. On other crops, Yukio Nagano and others used Rad-Seq SNP to mark 95 loquat germplasm data, and roughly divided loquat into three categories^26^.Esnart and other genetic structure results based on phylogenetic tree, and 81 cajanus cajanus were divided into three groups^23^.To sum up, there are few clustering results in this experimental group. The main source of this experiment material is that the germplasm screened out by field cold resistance evaluation is cold resistance germplasm, which leads to the lack of differences among genotypes and the lack of clustering results. In addition, the regions planted before screening are close to each other and have little geographical difference, which also leads to less clustering results.

### Construction of core germplasm bank

The primary goal in constructing core germplasm is to preserve the genetic information and structure of the original germplasm while maintaining its diversity with minimal samples. The sampling ratio for different plant core germplasm banks varies from 5 to 40%, without a unified standard^27^. Sun Yong et al.^15^ suggest that the sampling ratio depends on the original germplasm capacity and genetic diversity. Livia et al.^2^ analyzed 1117 rubber tree resources and created a core germplasm bank of 99 materials, accounting for 8.86% of the original population. An et al. ^16^selected 4462 wild rubber trees and screened 279 core samples, representing 6.25% of the original sample size. Fang et al. ^17^analyzed 2000 rubber tree resources and established a core germplasm bank of Weikehan rubber tree, comprising 27 germplasms, or 9.34% of the original resources. In our study, 21 samples were chosen from 195 rubber tree germplasms, making up 10.77% of the original sample trees. It's evident that as the number of rubber trees increases in the original germplasm bank, the sampling ratio in the core germplasm bank decreases.

The construction of core germplasm banks involves several methods, including grouping, stepwise clustering, and complete randomization. Grouping is used to ensure both representativeness and genetic diversity under different conditions, while also minimizing the impact of environmental factors. Common criteria for grouping include geographical origin, agronomic traits, breeding systems, and genetic markers. Stepwise clustering aims to identify materials with high genetic redundancy and eliminate them based on the results of each classification, while the completely random method strictly follows the principle of equal opportunity. In this particular experiment, involving the evaluation of early field cold resistance in rubber tree germplasm, the relatively small phenotypic differences and potential for significant human error make the grouping method unfeasible. As a result, the study utilized the clustering method, dividing the experiment into two groups: one with 169 materials and the other with 26 materials. However, the uneven distribution of materials meant that using the complete random method would not accurately extract the required materials, risking the loss of important germplasm resources due to the randomness of sampling. Therefore, clustering was employed to construct the core germplasm bank, aiming to reduce bias and ensure the representation and scientific validity of the selected materials.

The basic requirement for constructing core germplasm is to preserve the genetic information and structure of the original germplasm, with the primary consideration being the selection of methods, in order to maintain the genetic diversity of the original germplasm resources to the greatest extent possible with the minimum number of germplasm. This experiment used clustering method and five sampling ratios to construct a core germplasm bank, comparing He, PIC, pi values, and screening sampling ratios with the original germplasm retention rate. Finally, a 10% sampling ratio was selected to construct a core germplasm bank. The key to building core germplasm is to maintain the genetic diversity of existing germplasm resources to the greatest extent with the minimum number of germplasm^27^. The sampling ratio of different plant core germplasm banks ranges from 5 to 20%, and there is no unified standard^27^. Sun Yongqiang et al.^15^ believe that the size of the sampling ratio is mainly determined by the original germplasm capacity and genetic diversity. When the original sample size is small and the genetic diversity is high, the sampling ratio of the core germplasm bank can be relatively large. Conversely, the sampling ratio should be correspondingly reduced. Livia et al.^2^ analyzed 1117 rubber tree resources and constructed a core germplasm bank consisting of 99 materials, accounting for 8.86% of the original population; An Zewei^16^ selected 4462 wild rubber trees as the research object and screened 279 core samples, accounting for 6.25% of the original sample size; Fang et al.^17^ analyzed 289 rubber tree resources and constructed a core germplasm bank of rubber tree Weikehan germplasm, which includes 27 germplasm, accounting for 9.34% of the original resources. In this study, 21 samples were selected from 195 rubber tree germplasm to construct the core germplasm bank, accounting for 10.77% of the original sample trees. It can be seen that as the number of rubber trees in the original germplasm bank gradually increases, the sampling proportion in the core germplasm bank gradually decreases, which to some extent supports the research conclusion of Sun Yongqiang et al.^15^ The methods for constructing core germplasm banks include grouping, stepwise clustering, and complete randomization. Grouping is first to reflect representativeness and genetic diversity under different conditions, and secondly to minimize the impact of environmental factors. Common grouping criteria include geographical origin, agronomic traits, breeding systems, and genetic markers. Different crops should be grouped based on the characteristics of the collected resources. The basic principle of stepwise clustering is to identify materials with high genetic redundancy and eliminate them based on the results of each classification. The completely random method is a process that strictly follows the principle of equal opportunity. The experimental materials for this experiment are germplasm selected from early field cold resistance evaluation, all of which are planted in the germplasm resource garden of the rubber plantation at the Zhanjiang Experimental Station of the Chinese Academy of Tropical Agricultural Sciences. The distribution is concentrated, and the phenotypic differences of rubber trees are small, which can easily lead to significant human errors when distinguishing. Therefore, the grouping method is not feasible in this experiment. The clustering method divides this experiment into two groups. The first group includes 169 materials, while the second group includes 26 materials. The distribution of materials is extremely uneven. If the complete random method is used in this experiment, it will not be possible to accurately extract the required materials, which may lead to the loss of some important germplasm resources due to the randomness of sampling. Therefore, in order to make the core germplasm more representative and scientific, clustering is used to reduce bias. In summary, this experiment used clustering method and five sampling ratios to construct the core germplasm library, compared *He*, *PIC*, *pi* values, and the sampling ratio for screening the retention rate with the original germplasm. Finally, a 10% sampling ratio was selected to construct the core germplasm library, indicating that the constructed core germplasm library has good representativeness. In practical breeding work, the germplasm from the core germplasm library can be given priority consideration as breeding materials.

### Fingerprint construction

DNA fingerprinting can directly reveal the genetic differences between ornamental plant varieties at the DNA level, with high accuracy, polymorphism, and stability. It is not affected by the environment or its own growth status, and can provide a direct and effective theoretical basis for the improvement and protection of varieties. At present, fingerprint maps of cotton^28^, moss^29^, and honeysuckle^30^ have been constructed, providing important basis for the breeding of new plant varieties. In this study, the DNA fingerprint library of 195 rubber trees was constructed by 20 highly differentiated SNP, assigning each test material a unique code.The DNA fingerprint library can be directly used for the selection of rubber tree hybrid parents, better exerting heterosis, and promoting the breeding process of rubber trees. However, the existing fingerprint still has room for improvement. In the future, with the progress of breeding technology and the possibility of cold-resistant germplasm, more SNP markers need to be integrated.

## Methods and material

### Material

The 195 rubber tree accessions were chosen from an early field cold resistance evaluation and were planted at the Zhanjiang Experimental Station of the Chinese Academy of Tropical Agricultural Sciences.The location, on the northern edge of the tropics, experiences a monsoon climate with plentiful sunlight (1816.8–2073.5 h of sunshine per year), sufficient warmth (8309.29–8518.8 °C accumulated annual temperature), and copious rainfall (1396.3–1759.4 mm of rainfall per year). Each material was planted with three adjacent plants and three replicates, with a row spacing of 3 m between each two plants and 3 m between rows.

### DNA extraction and detection

To extract the DNA from the leaves of the rubber tree genome, we employed the method described by Murray et al.^17^. We used 1% agarose gel electrophoresis to assess the purity and concentration of the extracted DNA. The stock solution was then stored at − 20 °C for future use. For the detection, we required that agarose gel electrophoresis demonstrate a clear and intact main band of gene DNA, with no signs of degradation or RNA pollution. Additionally, the OD260/280 ratio, as measured by Nanodrop, should be between 1.8 and 2.0, indicating a lack of protein or visible impurity contamination.

### Primer design

To obtain SNP data for the rubber tree, we retrieved relevant data from published article databases^3^. After screening 548 SNP loci with a Minor Allele Frequency (MAF) > 0.3 and deletion genotype ratio < 0.5, we ensured an even distribution of these loci at a distance of 2 Mb. We then used e-PCR software to assess the amplification specificity of each primer pair, resulting in 335 pairs of specific primers that targeted 335 SNP loci. To ensure the discriminatory power of all samples, we selected 40 SNP loci for further analysis. Using Primer3 software (version 2.5.0)^31^, we designed primers with specific parameters: a primer sequence length ranging from 17 to 32 bp, a Tm value between 60 and 64 °C (with an optimal value of 62 °C), a product size not exceeding 500 bp, and sequencing reads that could cover the target site. For each target site, we designed multiple primer pairs and used e-PCR software (version 2.3.12) to test their amplification specificity. We ultimately selected the primer pair that could specifically amplify the target site. In this study, we used 40 primers to analyze the genetic diversity of 196 rubber tree materials, and the primer information is summarized in Supplementary Table 5. Choose 20 primer pairs for constructing fingerprints by applying the principle of distinguishing all samples.

### Library construction and quality control

To ensure the quality of library construction, genomic DNA samples undergo testing to confirm their suitability. Once the samples pass the genomic DNA detection phase, primers are mixed according to the experimental objectives and site-specific information, using the genomic DNA as a template. Subsequently, the target region is amplified using KAPA2G Fast Multiplex Mix, and sequencing adapters are added via secondary PCR. The resulting products are then pooled and purified using AMPure XP Beads. Once the library construction is complete, its quality is evaluated. Initial quantification is carried out using Thermo Life Qubit 3.0, while Agient 2100 is used to check the insert size of the library. The subsequent experimental steps can only proceed if the insert size meets expectations and there is no evidence of cross-contamination. Finally, the German ANALYTIKJENA QTOWER real-time fluorescence quantitative PCR instrument is employed to accurately determine the effective concentration of the library. A qualified library is defined as having an effective concentration greater than 2 nM.

### Machine sequencing and mutation detection

Pooling the library based on the target offline data volume and Paired end 150 bp (PE150) sequencing using Illumina HiSeq platform.

In this study, we pooled libraries based on the target offline data volume and used the Illumina HiSeq platform with Paired end 150 bp (PE150) sequencing. After comparing and analyzing the Reads with the reference genome, we obtained the BAM file. We used the HaplotypeCaller module in GATK (version 3.7) to generate gvcf files for each sample^32^. Subsequently, we employed the GenotypeGVCFs module to conduct mutation detection on all samples together. Our main focus was on inter-sample variation. Therefore, we filtered the initial variant results based on specific criteria: sequencing depth ≥ 20 for each sample, genotype deletion ≤ 10% across all samples, and allele frequency ≥ 5%. Sample 161 had a 100% deletion rate and was excluded from further analysis.

### Data statistics and analysis

In SNP markers, each locus exhibits three genotypes (0/0, 0/1, 1/1) and deletion values. The values of 0, 1, and 2 indicate different allelic polymorphisms. A value of 0 signifies the 0/0 homozygous genotype, 1 represents the 0/1 heterozygous genotype, and 2 indicates the 1/1 homozygous genotype. Deletion values of 9 are also recognized.

We use the online tool (http://w3.georgikon.hu/pic/english/default.aspx) to facilitate the calculation of expected heterozygosity (*He*) and polymorphism information content (*PIC*) values. This program, PICcalc, can calculate these values from manually uploaded allelic frequencies or from a givenfle containing binary data^33^. Using VCFtools software to calculate nucleic acid diversity (pi), and refer to Peter for data input and statistical methods^34^. For population genetic structure analysis, Structure (version 2.3.4) software is utilized. The method is a model-based clustering technique that utilizes genotype data to deduce population structure and allocate individuals into specific groups. During the process, an admixture model with independent allele frequencies was employed to estimate potential groups (K) ranging from 1 to 15. In order to ensure robust subpopulations, other parameters were set at elevated levels, including a 100,000 burn-in length followed by 100,000 Monte Carlo Markov Chain (MCMC) iterations, with each K being run five times. The "Structure Harvester" program (available at http://taylor0.biology.ucla.edu/structureHarvester/) was utilized to identify the plateau of ΔK in order to determine the optimal K-value. A Q-matrix was derived from the membership probability of each accession^35^. Principal Component Analysis (PCA) analysis is conducted using PLINK^36^, with reference to Eran's^37^ operating method Construct a phylogenetic tree using the neighbor-joining method in MEGA7 software^38^ and visualize it using ggtree^39^. The original germplasm resources undergo clustering analysis using MEGA7 (version 7.0) software. Five sampling ratios of 10%, 20%, 30%, 40%, and 50% are utilized. Genetic diversity parameters are calculated using PICcalc software, and the retention rate of these parameters is calculated to assess the representativeness of the core subsets at various sampling ratios.

To generate a fingerprint map, the genotype is converted into a digital fingerprint code. This fingerprint code is then used to create a two-dimensional code that serves as the basis for constructing a DNA fingerprint map in the form of a two-dimensional code.

### Supplementary Information

Supplementary Tables.

## Data Availability

Data are available from the corresponding authors on reasonable request.
